# Increased Nek1 expression in Renal Cell Carcinoma cells is associated with decreased sensitivity to DNA-damaging treatment

**DOI:** 10.18632/oncotarget.2005

**Published:** 2014-05-25

**Authors:** Yumay Chen, Chi-Fen Chen, Rosaria Polci, Randy Wei, Daniel J. Riley, Phang-Lang Chen

**Affiliations:** ^1^ Department of Medicine, Division of Endocrinology, University of California at Irvine; ^2^ Department of Biological Chemistry, University of California at Irvine; ^3^ Department of Medicine, Division of Nephrology, The University of Texas Health Science Center at San Antonio; ^4^ Department of Surgery, The University of Texas Health Science Center at San Antonio

**Keywords:** Renal Cell Carcinoma, VDAC1, Nek1

## Abstract

Renal cell carcinoma (RCC) is a heterogeneous disease with resistance to systemic chemotherapy. Elevated expression of multiple drug resistance (MDR) has been suggested to be one of the mechanisms for this resistance. Here, we provide an alternative mechanism to explain RCC's resistance to chemotherapy-induced apoptosis. Never-in mitosis A-related protein kinase 1 (Nek1) plays an important role in DNA damage response and proper checkpoint activation. The association of Nek1 with the voltage-dependent anion channel (VDAC1) is a critical determinant of cell survival following DNA-damaging treatment. We report here that Nek1 is highly expressed in RCC tumor and cultured RCC cells compared to that of normal renal tubular epithelial cells (RTE). The association between Nek1 and VDAC1 is genotoxic dependent: prolonged Nek1/VDAC1 dissociation will lead to VDAC1 dephosphorylation and initiate apoptosis. Down-regulation of Nek1 expression in RCC cells enhanced their sensitivity to DNA-damaging treatment. Collectively, these results suggest that the increased Nek1 expression in RCC cells maintain persistent VDAC1 phosphorylation, closing its channel and preventing the onset of apoptosis under genotoxic insults. Based on these results, we believe that Nek1 can serve as a potential therapeutic target for drug development in the treatment of RCC.

## INTRODUCTION

Renal cell carcinoma (RCC) is the most common and lethal type of kidney cancer in adults. When a tumor is confined to the kidney, surgical approaches remain as a curative treatment. However, one-third of the patients have metastatic forms and require systemic chemotherapy [1]. Unfortunately, RCC is a chemotherapy-resistant tumor [2, 3]. A path leading to complete and durable responses face many challenges because the heterogeneous nature of RCC contributes to the increase in tumor incidents and mortality. Thorough understanding of the molecular signature and the chemotherapy resistance nature of RCC remains an important goal in the identification of targets for effective treatment.

Intrinsic apoptotic pathways that respond to cytotoxic stress, including DNA damage, activate a hierarchical series of caspases that disassemble cells without inciting inflammation in bystander cells [4-7]. The onset of apoptosis is initiated by the release of cytochrome C from mitochondria; after its release into cytoplasm, it activates Apaf-1 (apoptosis protease activating factor) which cleaves a series of caspases that ultimately dismantle the cell by breaking down cell-cell contacts, cytoskeletal elements, and nuclear structures [8-10]. Permeabilization of mitochondria is seminal for the regulation of apoptosis induced by cytotoxic stress and is regulated by the mitochondrial permeability transition pore, composed of the outer mitochondrial membrane protein VDAC1 (a.k.a. porin), the inner mitochondrial membrane protein ANT (adenine nucleotide translocator), and the inner membrane associated protein cyclophilin D [11-15]. One of the key regulators for VDAC1 channel is NIMA-related protein kinase 1 (Nek1) [16, 17].

Nek1 is important in the regulation of cell survival, in part by monitoring the DNA damage response as well as the apoptosis pathway via its interaction with VDAC1 [17-19]. In the basal state, Nek1 phosphorylates VDAC1, which maintains the mitochondrial membrane potential (MMP). When the expression of Nek1 is down-regulated, cells easily lose the S193 phosphorylation of VDAC1 (Nek1 phosphorylation site) when injured, as by a DNA damaging agent, and die even with a DNA damage dose that would not kill identical cells with a normal amount of Nek1. Taking the roles of Nek1 in DNA repair pathways and apoptosis suggests that Nek1 functions in defense against oxidative stress and/or DNA damage, and that relatively trivial environmental injury may cause unscheduled and excessive cell death in Nek1-deficient cells. Two strains of mice, Kat and Kat2J, without functional Nek1 developed polycystic kidney disease (PKD) and other systemic manifestations [20, 21]. In PKD, one of the key factors in development and progression to renal failure is unregulated cell death in renal tubular epithelial cells. Different expression levels of the wild-type Nek1 in various cell types may account for a disparity in survival from environmental injury. Accordingly, higher expression level of Nek1 could be a contributing factor for the chemotherapy resistance found in many malignances, especially in RCC.

In this report, we found that Nek1 expression is increased in RCC compared to RTE. Association of NEK1, VDAC1 and subsequent VDAC1 S193 phosphorylation status is regulated according to different levels of genotoxic stress. Downregulation of Nek1 expression by small interference RNA increases sensitivity of RCC to DNA-damaging chemotherapeutic agents.

## RESULTS

### Nek1 is overexpressed in established RCC lines

**Figure 1:**
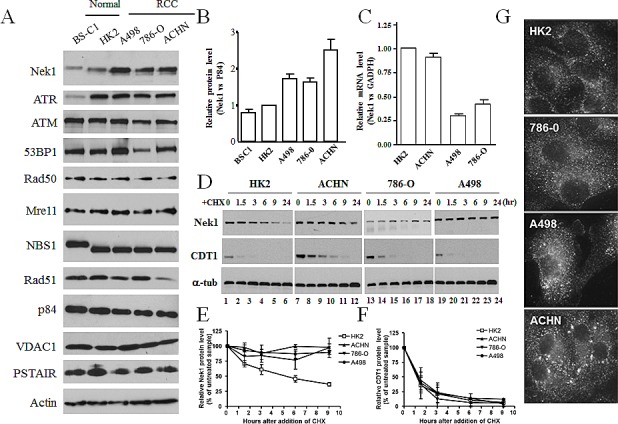
Expression and subcellular localization of Nek1 in renal cell carcinoma cell lines A & B. Nek1 expression in renal cell carcinoma cell lines. Total cell lysates were prepared from a normal monkey diploid kidney cells (BS-C1), normal human renal diploid cells (HK2), and three human renal cell carcinoma cell lines (A498, 786-O, and ACHN). Protein expression was analyzed by Western blotting using several antibodies. To control for total protein loading, p84, PSTAIR, VDAC1, and actin, were analyzed from the same samples. Nek1 expression in HK2 cells was normalized and given a value of 1. The relative amounts of Nek1 expressed in BS-C1, A498, 786-O, and ACHN cells were then expressed as fold-difference relative to HK2. Results represent analysis and quantification of three independent blots (means s.e.m.). C. Expression of Nek1 mRNA in RCC cells. Total RNA was purified from RCC cells. After reverse transcription, the resulting cDNAs were analyzed for Nek1 and GADPH expression using real-time PCR. Nek1 expression was normalized to GADPH, and expression in HK2 cells was again given a value of 1. D-F. Nek1 protein is stable in RCC cells. D. RCC and HK2 cells were treated with 100ug/ml cycloheximide for indicated time and level of Nek1 protein was detected by anti-Nek1 antibodies. Level of CDT1 was determined as control for the effect of cycloheximide. Nek1 (E) and CDT1 (F) protein levels were normalized with tubulin and expression as 100% at time zero. The protein amount was then plot against time as a mean from three independent experiments. Nek1 protein was stable in RCC cells. G. Subcellular localization of Nek1 in renal cell carcinoma. Cells in the exponential growth phase were grown on coverslipes, fixed with 4% neutral buffered formalin and then incubated with rabbit polyclonal anti-Nek1 primary antibodies and fluorescence-tagged anti-rabbit IgG secondary antibodies. Nek1 is localized primarily in the cytoplasm of all cells.

Since Nek1 expression level is a critical factor that modulates cellular responses to DNA damage, we investigated the Nek1 expression in RCC and normal diploid cells. An affinity purified rabbit anti-Nek1 antibody was used to examine Nek1 protein expression by Western Blotting analysis in the RCC cell lines: A498, 786-O and ACHN. In the three RCC cell lines, there is a 2 to 3 fold increase in the Nek1 protein compared to normal RTE (Figure 1 A, B). Given that Nek1 is a protein kinase and that its expression is up-regulated in response to stress, we also examined the expression of other protein kinases and DNA damage response proteins in these RCC cell lines. Unlike Nek1, all other proteins examined showed no significant or consistent differences between RCC and normal cells (Figure 1A). VHL-associated transcription regulation (VHL/HIF pathway) is known to be aberrant in a subset of RCC. In order to determine if the elevated expression of Nek1 protein in RCC is due to transcriptional upregulation, we analyzed Nek1 mRNA using real-time PCR analysis. Nek1 mRNA is significantly down-regulated in two RCC lines (A498 and 786-O) and slightly down-regulated in ACHN cells (Figure 1C). The gene-encoding VHL is mutated in A498 and 786-O cells but it is wild-type in ACHN cells. This difference between the established RCC cells suggests that the VHL/HIF pathway could regulate Nek1 at the transcriptional level. Nonetheless, Nek1 protein level is independent of the status of VHL since there is a 2-3 fold increase in Nek1 protein in both VHL mutant (A498 and 786-O) and VHL wild-type (ACHN) cells compared to normal cells (Figure 1A, B).

To further investigate the potential mechanism of the up-regulated Nek1 protein expression in RCC cells, we examined the Nek1 protein turnover rate in the established RCC cells (Figure 1D). Following the addition of cycloheximide, cellular lysate was prepared at different time points and Nek1 protein level was examined. Nek1 level was diminished quickly after cycloheximide treatment in HK2 cells, but all 3 RCC cells shown no deceased in Nek1 protein level, even after 24 hours. In all 4 cell lines, CDT1 were degraded quickly after cycloheximide treatment suggests that the protein synthesis were blocked by cycloheximide in all 4 cell lines (Figure 1E&F). The result suggested the up-regulated Nek1 protein level in RCC cells is due to a failure of protein degradation mechanism for Nek1.

Altered gene expression or localization has been observed frequently in cancer cells. To investigate the state of Nek1 in RCC cells in more detail, we examined the subcellular localization of Nek1 in RCC cell lines by immunostaining. When cells were cultured in regular, unstressed conditions, Nek1 was localized in the cytoplasm by indirect immunocytochemical staining (Figure1G). The expected cytoplasmic staining of Nek1 in the RCC and normal tubular epithelial cell lines also suggested that the upregulation of Nek1 in RCC cell lines is not due to peculiar subcellular localization. Next, to ensure that the function of Nek1 is intact in RCC cells, we also examined the formation of Nek1 nuclear foci in RCC cells after DNA damage and its kinase activity. Upon DNA damage in RCC cells, Nek1 kinase activity was up-regulated while Nek1 relocated to the nuclei DNA damage sites, as it does in normal diploid cells [data not shown and [22]]. These results suggested that the up-regulated Nek1 in RCC cells is functional.

### Nek1 is up-regulated in human renal cell carcinomas

**Figure 2:**
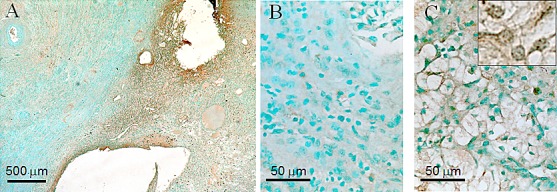
Overexpression of Nek1 in renal cell carcinoma Formalin-fixed, paraffin sections of renal cell carcinoma kidneys were probed with purified, rabbit anti-Nek1 primary antibodies, and detected by biotin-labeled secondary antibodies and the Vector ABC system for immunohistochemical analysis. At low magnification (25X) (A), Nek1 expression is markedly higher in the tumor (right side) compared to surrounding normal kidney (left side). Higher power magnification (40X) shows subcellular localization of Nek1 in normal tubular epithelial and glomerular cells (B), and clear cells (C). The nuclear punctate dots staining pattern was observed in 100X magnification (C, insert). Similar staining pattern (much more Nek1 expression in RCC than in surrounding normal kidney parenchyma) was observed in 5 individual tumor samples (all Stage 1 tumors).

The RCC cell lines analyzed above have been cultured in vitro for years, and the upregulation of Nek1 observed could be acquired from multiple passages in vitro. To confirm that Nek1 is up-regulated in human RCC, we examined Nek1 expression of human specimens from a tissue bank. Affinity purified rabbit anti-Nek1 antibody was again used to perform the immunohistochemistry (IHC) analysis. The specificity of anti-Nek1 antibodies for IHC has been well established in many ways, including the observation that it does not recognize any protein in Nek1-null tissue [22]. Immunohistochemistry analysis on formalin-fixed, paraffin-embedded tissue from 5 samples of stage I renal clear cell carcinoma showed that Nek1 expression is significantly increased in neoplastic cells compared to the adjacent normal renal tubular epithelial cells, which have very little Nek1 expression (Figure 2A). In previous studies [18, 22], we have shown that Nek1 is located in the cytoplasm of the undamaged cells. Upon DNA damage to the cells, Nek1 translocates to nuclei at sites of DNA damage, in a punctate staining pattern. Using high power magnification, we observed that Nek1 is localized to the cytoplasm in RCC clear cells, but also in nuclei as punctate dots (Figure 2B, C). The nuclear staining at discrete foci (Figure 2C, insert) suggests that Nek1 responds to DNA damage in the RCC clear cells.

### Renal carcinoma cells are resistant to the effects of genotoxic treatments

**Figure 3:**
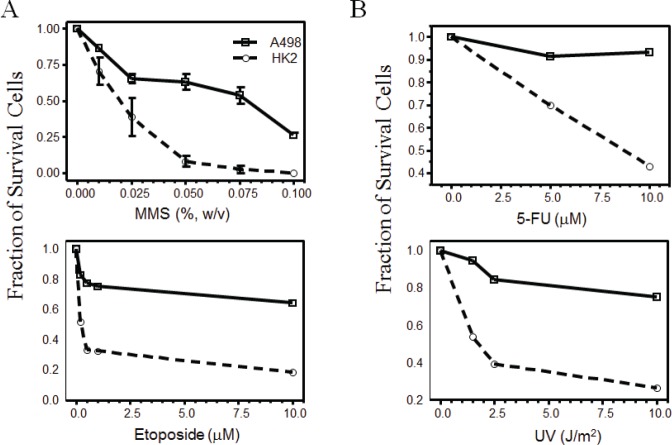
Renal cell carcinoma are resistant to genotoxic agents HK2 and A498 cells in exponential growth phase were plated out at a density of 2x10^5^ cells per 60-mm dish, 16 hours before the genotoxic treatment. Cells were treated with the indicated doses of methylmethane sulfonate (MMS, a DNA alkylating agent), 5-FU, etoposide (a topoisomerase II inhibitor), or ultraviolet radiation (UV). Twenty-four hours after treatment, live cells excluding trypan blue were counted and expressed as the fraction of surviving cells compared to mock-treated cells. Each data point represents mean s.e.m. from two independent experiments.

Nek1 has been shown to regulate mitochondrial-mediated cell death through its interaction with and phosphorylation of the mitochondrial outer membrane protein, VDAC1. VDAC1 and the mitochondrial permeability transition pore open easily in the absence of Nek1, which in the basal state, seems to phosphorylate VDAC1 and keep the channel closed. To further investigate the effect of Nek1 overexpression in RCC cells' resistance to genotoxic chemotherapy and radiation, we hypothesize that a decrease in the RCC's expression of Nek1 protein would sensitize these cells to genotoxic agents by reducing the threshold for the induction of cell death. First, we performed dose-response analyses to determine the sublethal and lethal doses for normal kidney diploid cells and RCC cells by treating HK2 transformed diploid human renal tubular epithelial cells and A498 RCC cells with different genotoxic agents and then examining their survivability. For genotoxic drug dosing, cells were first treated with different agents at varying doses for one hour. At the end of the 1-hour treatment, the drugs were either neutralized and/or washed away, and fresh media was added to the cultures. The A498 cells consistently showed higher tolerance to the given genotoxic treatment compared to HK2 cells (Figure 3). For example, the sublethal dose of methylmethane sulfonate (MMS) is 0.025% and the lethal dose 0.05% (W/V) for HK2 cells, while the sublethal dose is 0.075% and the lethal dose higher than 0.1% for A498 cells. Similar results were also observed in 786-O and ACHN cells (data not shown).

### Dosage-dependent interaction between Nek1 and VDAC1 in response to genotoxic treatments

**Figure 4:**
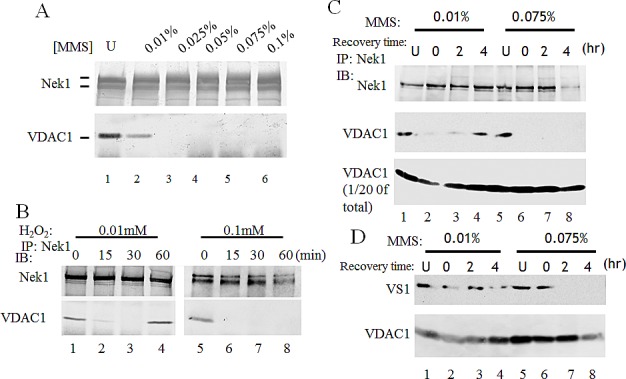
Interaction between Nek1 and VDAC1 depends on the dose of the genotoxic agent A. HK2 cells were treated with the indicated doses of MMS for 1 hour. Cells were washed twice with PBS and lysed. Clarified lysates were immunoprecipitated with anti-Nek1 antibodies. The resulting immune complexes were separated on a gel and analyzed by Western blotting with anti-Nek1 or anti-VDAC1 antibodies. Nek1 and VDAC1 dissociated after a high dose of methylmethane sulfonate (MMS) treatment (lanes 3 to 6). B & C. The association between Nek1 and VDAC1 is reversible with sublethal dose of a genotoxic agent, but irreversible after a lethal dose. B. HK2 cells were treated with H_2_O_2_ at the indicated concentrations for the specified time periods. Soon after H_2_O_2_ treatment, Nek1 and VDAC1 dissociate (lanes 2 and 6). Sixty minutes after treatment with a sublethal dose of H_2_O_2_ (0.01 mM), Nek1 and VDAC1 re-associate (lane 4), but they do not re-associate after an ultimately lethal dose (0.1 mM, lane 8). C. HK2 cells were treated with MMS at either 0.025% (W/V) (sublethal dose) (lanes 1 to 4) or 0.075% (W/V) (lethal dose, lanes 5 to 8) for one hour. One hour after treatment, MMS was neutralized by sodium thiosulfate, and cells were washed twice with PBS and re-fed fresh media. At the indicated times, cells were harvested for analysis. Nek1 and VDAC1 dissociated after MMS treated (lanes 2 and 6). Nek1 and VDAC1 re-associated 4 hours after a sublethal dose of MMS (lane 4), but not after an ultimately lethal dose in the same type of cells (lane 8). Mock-treated cells (U, lanes 1 and 5) were used as controls. D. The Nek1-dependent, S193 phosphorylation of VDAC1 was lost after an ultimately lethal dose of MMS. HK2 cells were treated with MMS as described in C. Total HK2 cell lysates and VS1 antibodies were used to analyze VDAC1 phosphorylation at S193, a key Nek1-specific phosphorylation site [17]. After a sublethal dose of MMS, the Nek1-dependent S193 phosphorylation of VDAC1 is intact (upper panel, lanes 1 to 4), immediately after 4 hours. After an ultimately lethal dose of MMS, the pattern of VDAC1 phosphorylation on S193 is different. Immediately after the lethal treatment, the S193 phosphorylation is intact (lane 6). This Nek1-dependent phosphorylation of VDAC1 is rapidly lost, however, and is never recovered, up to 4 hours later (lanes 7 and 8).

As shown in our previous reports, Nek1 interacts and phosphorylates VDAC1 at S193 in untreated normal cells [16, 17]. This interaction and phosphorylation of VDAC1 by Nek1 is important for cell survival. Upon a lethal dose of UV irradiation, Nek1 and VDAC1 dissociate from each other, and dephosphorylation of VDAC1 at S193 precedes cell death. To know whether the Nek1-VDAC1 interaction is modulated in response to different doses of genomic insults, we determined the dose required to induce dissociation of Nek1 from VDAC1. HK2 cells were treated with different doses of the alkylating agent, MMS. At the end of 1-hour treatment with sublethal or lethal doses of MMS as determined in Figure 3, cells were harvested and Nek1-VDAC1 immune complexes were examined by co-immunoprecipitation (co-IP) (Figure 4A). A sublethal dose of 0.025% for MMS was required to induce dissociation of Nek1 from VDAC1 in HK2 cells. If the phosphorylation and interaction with Nek1 is important for VDAC1's function and cell survival, then the dissociation of Nek1 from VDAC1 with sublethal treatment doses should be followed by re-association during the recovery phase, before seminal dephosphorylation of VDAC1 on S193 occurs. Re-association of Nek1 with VDAC1, however, would not be expected before VDAC1 dephosphorylates in the same cells treated with lethal doses of genotoxic agents. To test this possibility, HK2 cells were treated with a sublethal or lethal dose of different genotoxic agents and Nek1-VDAC1 complexes were examined at various time points during the recovery phase. Co-IP experiments performed in HK2 cells treated with either H_2_O_2_ (Figure 4B) or MMS (Figure 4C) demonstrated dissociation of Nek1 and VDAC1 one hour after either a sub-lethal or lethal dose of either genotoxic treatment. Re-association of Nek1 and VDAC1, however, identified by co-IP complexes, was detected only in the cells treated with sublethal doses. In the cells treated with lethal doses, Nek1-VDAC1 complexes were not detected. The Nek1-dependent phosphorylation status of VDAC1 following MMS treatment was also examined using VS1, phospho-VDAC1 (S193) antibodies. VDAC1 remains phosphorylated on S193 in the cells treated with a sublethal dose of MMS. In contrast, if the cells were treated with a lethal dose of MMS, VDAC1 S193 phosphorylation was lost during what should be the recovery phase from the injury (Figure 4D).

### Reducing Nek1 expression allows renal carcinoma cells to be more sensitive to genotoxic treatments

**Figure 5:**
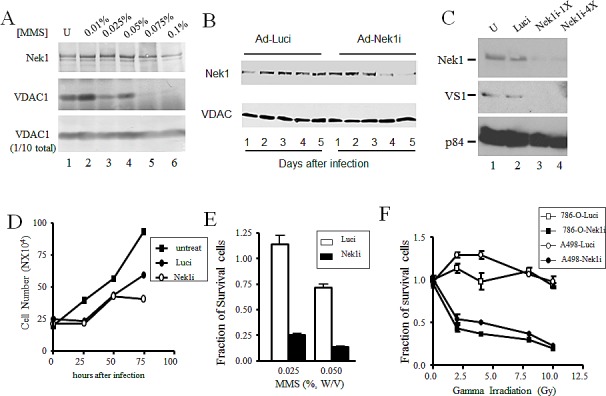
Downregulation of Nek1 expression allows RCC cells more sensitive to genotoxic agents A. Dose-response: compared to HK2 cells, a higher MMS dose is required in RCC cells to cause the dissociation of Nek1 from VDAC1. A498 cells were treated with the indicated doses of MMS for 1 hour. After washing three times with PBS, cells were lysed and Nek1-VDAC1 protein complexes were analyzed by Western blotting, as in Fig. 4. B. Downregulation of Nek1 expression in A498 cells by RNAi interference. Short inhibitory RNA, in an adenovirus-based DNA expression system, was used to silence expression of Nek1 in A498 cells. Days after infection of A498 cells with the siRNA constructs, separated proteins from cell lysates were probed for either the Nek1 expression (upper panel) or VDAC1 (lower panel). Nek1 expression was markedly reduced by day 4 (96 hours) after an infection with the Ad-Nek1i. Infection with a control construct to inhibit firefly luciferase (Ad-Luci) had no effect on VDAC1 expression. C. 72 hours after the infection, there was a loss of VDAC1 phosphorylation on S193 in A498 cells expressing Nek1i. Gel-separated proteins from cell lysates were probed for Nek1 expression (upper panel), VDAC1 phosphorylated on S193 (VS1, middle panel), and p84 (bottom panel, to control for loading). U, mock-infected cells; Luci, cells infected with Ad-Luci, used as a negative control; Nek1-1X, cells infected with a construct containing a single copy of the siRNA oligonucleotide specific for Nek1; Nek1-4X, cells infected with an otherwise identical construct containing 4 tandem repeats of the same siRNA oligonucleotide. D. Growth curve of cells after adenovirus infections. A498 cells were plated out at 1X10^5^ cells per 60mm dishes and infected with either Ad-Luci or Ad-Nek1i 24 hours later. Surviving cells (those excluding trypan blue vital dye) were counted every 24 hours and cell number was plotted. The adenovirus infections did not alter the cell growth during the first 60 hours, and Nek1i infected cells started to show no cell number increase after 72 hours (3 experiments). E. Increased sensitivity to the toxicity of MMS in cells expressing Nek1i siRNA. A498 cells were plated out and infected with either Ad-Luci or Ad-Nek1i. Sixty hours after the infection, the cells were either mock-treated or treated with 0.025% or 0.05% (W/V) MMS for 1 hour. After neutralization with sodium thiosulfate, the cells were washed three times with PBS and re-fed with fresh media. Twenty-four hours later, surviving cells were counted and expressed as fractions of identical, mock-treated cells (5 experiments). F. Greater sensitivity to ionizing radiation in cells with Nek1 expression downregulated. A498 and 786-O cells were infected with Ad-Luci or Ad-Nek1i. Sixty hours after the infection, the cells were irradiated with different doses of IR and surviving cells (those excluding the trypan blue vital dye) and were counted 24 hours later (5 experiments).

So far, we have demonstrated that the abundant expression of Nek1 seems to protect cultured RCC cells from drug- or radiation-induced, genotoxic apoptosis, at least in part through Nek1's interaction with and phosphorylation of VDAC1. This molecular mechanism may underlie the genotoxin-resistant nature of RCC. To test this possibility more directly, we examined the dose-dependent interaction between Nek1 and VDAC1 in RCC cells. A498 cells were treated with different doses of MMS for 1 hour and the Nek1-VDAC1 interaction was analyzed by co-IP. In contrast to diploid HK2 cells, A498 cells require a higher dose of MMS to induce the dissociation of Nek1 and VDAC1 (Figure 5A). If the high expression level of Nek1 accounts for stable Nek1-VDAC1 complexes detected in RCC cells treated with a high dose of genotoxic agents, then the downregulation of Nek1 expression to decrease the Nek1 in the cytosol in RCC cells might increase their respective sensitivity to genotoxic agents. To test this hypothesis, we employed RNAi technology to knock down Nek1 expression in two RCC cell lines, A498 and 786-O. To assure near 100% targeting, we expressed short-hairpin RNA (shRNA) targeting Nek1 in an adenovirus construct. First, we determined when Nek1 expression was significantly decreased after adenovirus infection. Cells were harvested every 24 hours after infection with adenovirus carrying shRNA targeting either Nek1 (Ad-Nek1i) or a control construct, luciferase (Ad-Luci). Cell extracts were then prepared and analyzed for Nek1 expression using Western blotting analysis. Ad-Nek1i specifically targeted Nek1 protein expression whereas control Ad-Luci, which targets luciferase, did not. Expression of Nek1 was significantly reduced by 96 hours after the infection. Downregulation of Nek1 expression had no effect on overall VDAC1 expression (Figure 5B), but it did markedly decrease the abundance of S193-phospho-VDAC1 (Figure 5C). Ad-Luci or Ad-Nek1i did not show any effect in cell growth and survival during the first 60 hours following virus infection (Figure 5D). To examine whether downregulation of Nek1 expression in RCC cells would sensitize them to treatments with genotoxic agents, we infected the cells with Ad-Nek1i or Ad-Luci. Sixty hours after the infection, the cells were either untreated or treated with different doses of genotoxic agents and analyzed 24 hours later. The fraction of surviving cells was determined by trypan blue exclusion as compared to the untreated cells. RCC cells infected with Ad-Nek1i were much more sensitive to the treatment of MMS (Figure 5E) or gamma irradiation (Figure 5F, Table 1). The control Ad-Luci had no effect on cell survival after treatment with either genotoxic agents. These results demonstrate that reducing Nek1 expression by RNAi mediated gene silencing can increase the sensitivity of targeted RCC cells to genotoxic agents.

**Table1:** Greater sensitivity to ionizing radiation in cells with Nek1 expression downregulated The data presented in figure 5F as the means + standard deviation of the fraction of surviving cells from 5 different experiments

Dose (Gy)	786-O-Luci	786-O-Nek1i	A498-Luci	A498-Nek1i
0	1.006+0.094	0.960+0.110	0.984+0.046	1.016+0.082
2	1.136+0.126	0.433+0.081	1.292+0.061	0.547+0.118
4	0.982+0.220	0.368+0.028	1.291+0.106	0.508+0.041
8	1.106+0.081	0.304+0.009	1.066+0.047	0.371+0.059
10	0.934+0.072	0.201+0.017	0.984+0.125	0.235+0.005
				N=5

## DISCUSSION

In this communication, we have reported that elevated Nek1 protein expression in RCC tumor and cultured RCC cells compared to that of normal RTE. Knocking down Nek1 expression in RCC cells renders them sensitive to DNA damaging treatments. These observations are consistent with the idea that Nek1 protect cells from oxidative stress and/or DNA damage. They also support the notion that Nek1 expression level is particularly critical in malignant RCC tumors, which often resist DNA-damaging therapeutic agents and in which hypoxia-induced oxidative injury and genomic instability are common. RCC is the first example to support the idea that significant overexpression of Nek1 might adjust cellular response to environmental insults.

Several NIMA-related protein kinases have been shown to exhibit altered expression level in neoplastic cells. They all play roles in cell cycle regulation and some in DNA damage responses. Among them, Nek2, a serine-threonine protein kinase, is the best-characterized one. Nek2 expression level is cell-cycle regulated [23]. Its interaction and phosphorylation of an important cell cycle regulator, HEC1, ensures the faithful chromosome segregation [24]. Elevated expression of Nek2 has been found in several different cancers, including breast cancer, testicular seminomas, cholangiocarcinoma, etc [25-27]. Furthermore, targeting Nek2 expression by RNA silencing gene expression has been shown to reduce the tumorigenic potential of targeted cancer cells [26, 28]. Unlike Nek2, Nek1 has dual serine/threonine and tyrosine kinase activity in vitro and its expression is not cell cycle-dependent. Both Nek1 and Nek2 are not required for cell survival. However, Nek1-deficient cells are markedly more sensitive to the lethal effects of DNA damage compared to otherwise identical cells expressing functional Nek1 [18, 22]. Lacking Nek1 expression, cells suffer unscheduled and excessive cell death in relatively trivial environmental insults. In contrast, our data here suggests that cells with Nek1 over-expression are more resistant to lethal effects of DNA damaging agents. Thus, to achieve a fine balance keeping cells from either apoptosis or accumulating harmful mutations, the Nek1 expression level needs to be tightly regulated.

The mechanism underlying Nek1 overexpression in RCC cells is not known. In our previous study, Nek1 expression is up-regulated in tubular epithelial cells after ischemic injury, before the cells undergo frank apoptosis, necrosis or prior to injury repair [29, 30]. Apparently, Nek1 expression levels in cells with high potential for errors in DNA damage repair or narrowly defined windows for proliferation and regeneration after injury becomes a critical issue. Insufficient Nek1 expression might result in loss of critical cells at critical time and leads to organ dysfunction or tumor formation [31]. Increased Nek1 expression leads to improved cell survival due to the failure of removing critically mutated cells. Candidates of those critical cells are renal epithelial cells and hepatocytes which detoxify waste and require frequent regeneration after injury.

RCC is well known for its chemotherapy resistance. Over the past few decades, intense research in the understanding of the molecular biology of RCC has identified tumor suppressor gene, von Hippel-Lindau (VHL), as an important molecule in RCC pathogenesis [32-34]. Inactivation of VHL has been found both in hereditary RCC as well as in most cases of sporadic clear-cell RCC. VHL encodes the VHL protein, a component of an E3 ubiquitin-ligase complex [35]. The immediate target of VHL-mediated protein degradation is the hypoxia-inducible factor 1A (HIF1A) [33, 36]. Thus, VHL plays an important role in regulating the hypoxic pathway through HIF1α. In hypoxic conditions, accumulated HIF1α binds to HIF1β to form the heterodimeric transcription factor, HIF, which in turn leads to the transcription of a wide range of downstream proteins [33]. Among those are VEGF, platelet-derived growth factor (PDGF) and transforming growth factor alpha (TGF-α) which all have active roles in tumor angiogenesis and progression [37]. Small molecule inhibitors or monoclonal antibodies for VEGF receptors and PDGF receptors have been developed for treating RCC [38]. Another important tumor suppressor gene, phosphatase and tensin homologue (PTEN), is also found frequently deleted or mutated in RCC [39]. PTEN functions to oppose phosphoinositide 3-kinase (PI3K) function, leading to the inactivation of AKT and mammalian target of rapamycin (mTOR) signaling [40]. Thus, the downstream mTOR pathway is also an ideal candidate for targeting treatment of RCC. The introduction of these new molecular targets has offered more options in the therapeutic strategy. Chemotherapy resistance remains an important clinical obstacle and research in this area may lead to new therapeutic options for patients with RCC.

The increased Nek1 protein in RCC is not due to transcriptional upregulation. In fact, the mRNA level of Nek1 is decreased in RCC cells and is correlated to VHL status. In the VHL mutant RCC cells, Nek1 mRNA is only 25% of the normal RTE-HK2, while it is only less than 15% reduction in RCC cells with the wild-type VHL. This increased Nek1 protein level does not seem to be regulated by VHL. The stability of Nek1 seems to be responsible for the increased Nek1 protein level in RCC. In HK2 cells, the half-life of Nek1 protein is around 3 hours. In RCC cells, the half-life of Nek1 is more than 24 hours. Which protein degradation pathway is responsible of Nek1 stability will need to be investigated further. The Nek1 protein kinase in the RCC cells appears to be functional, exhibits a proper subcellular localization and form IR-induced nuclear foci. In our previous reports, in cells treated with DNA damaging agents, Nek1 dissociated with VDAC1, and VDAC1 lost its Nek1-specific phosphorylation [17]. Nek1 regulates VDAC1 channel opening and closing through phosphorylation as examined by atomic force microscope (AFM) [16]. Here we show that this dissociation between Nek1 and VDAC1 and the loss of specific Nek1 phosphorylation on VDAC1 is genotoxic dosage-dependent. In cells treated with sublethal dose of genotoxic agents, Nek1 and VDAC1 temporarily dissociated from each other and the re-association occurred before VDAC1-S193P became dephosphorylated. However, in cells treated with a lethal dose of genotoxic agents, VDAC1 became dephosphorylated and was unable to re-associate with Nek1. In Nek1-overexpressed RCC cells, higher doses (often lethal to normal cells) of DNA damaging agents were needed to induce the dissociation between Nek1 and VDAC1. By knocking down the Nek1 expression using small RNA interference, RCC cells became hypersensitive to genotoxic agents when compared to control group. This observation opens a new window for developing a potential treatment for RCC.

The current treatments for RCC are surgical removal for all stages of RCC. In addition to surgery, several other treatments will be given to the patient, such as radiation therapy, arterial embolization, biological therapy or targeted therapy [41-43]. All the treatments rely on early diagnosis and surgical resection as the mainstay of RCC's treatment. Targeting proteins involved in both DNA repair pathway and apoptosis has been proposed for drug development in the treatment of chemo-resistant tumors. Among them, cell cycle regulatory protein, p21, has been considered a potential target molecule for RCC. Inhibition of p21 translation by antisense oligodeoxynucleotide or siRNA has shown to sensitize tumor cells to DNA damaging agents. For its role in both proliferation and differentiation, specific targeting in RCC will be needed for p21-mediated chemotherapy in order to avoid damage to normal kidney cells as well as other organs.

The increased Nek1 protein expression in VHL wild-type cells suggests that Nek1 expression is not regulated by the VHL pathway at the protein level. Unlike p21, which is accumulated in the cytosol of PTEN-negative RCC cells, the function and subcellular localization of Nek1 is not altered in RCC cells. While Nek1 mRNA is down-regulated in RCC, the protein level increased due to the longer half-life. This observation suggests that a protein degradation program is altered in RCC and their activity is not regulated by the VHL pathway. Simple inactivation of Nek1 expression by small interference RNA in RCC cells increased their sensitivity to DNA-damaging agents. The results presented in this report extended our understanding of the roles of Nek1 in cell survival and apoptosis in response to DNA-damaging agents. The fact that Nek1 levels determine cellular tolerance to DNA-damaging agent treatment might begin to explain the underlying mechanism of chemo-resistance observed in cancer cells. The expression level of Nek1, thus, becomes a key survival factor for a given cell in its tolerance against DNA-damaging agents.

## MATERIAL AND METHODS

### Cell Culture

Human ACHN, A498, and 786-O renal carcinoma cells, and HK2 human proximal renal tubular epithelial cells were obtained from the American Type Tissue Collection (Rockville, MD) and cultured in DMEM/F12 media containing 10% fetal bovine serum and antibiotics.

### Antibody

Anti-Nek1 and VS1 [anti-phospho-VDACl (S193)] antibodies were described [17, 22]. Anti-p84 mAb 5E10, anti-ATM (2C1), anti-ATR were purchased from Genetex (Irvine, CA). Anti-VDAC1 mAb2 and mAb3 was purchased from Calbiochem (La Jolla, CA). Anti-53BP1, Mre11, NBS, Rad51, PSTAIR, and Actin antibodies were purchased from Cell Signaling (Danvers, MA). Anti-CDT1 antibodies were purchased from Bethyl Laboratories (Montgomery, TX). Anti-tubulin (DM1A) antibodies were purchased from Sigma-Aldrich (St. Louis, MO). Fluorochrome-conjugated secondary antibodies (Alexa-Fluoro 488 for green) were purchased from Molecular Probes, Inc. (Eugene, OR), and horseradish peroxidase-based secondary antibodies from Vector Technologies (Burlingame, CA).

### Immunoprecipitation and Western blot Analysis

Cells were lysed in Lysis 250 buffer and subjected to freeze/thaw (LN2/37°C) and clarified by centrifugation. The supernatants were diluted with equal volume of Lysis 0 and used for immunoprecipitation as described [44]. Mouse polyclonal anti-Nekl antisera (1 μl) or purified mouse polyclonal anti-Nekl antibody (1 μg) was used for immunoprecipitation from each supernatant.

### Immunohistochemistry

Human biopsy samples obtained at the time of organ implantation/revascularization or removal. The specimens were fixed overnight in 10% neutral buffered formalin. After progressive dehydration and embedding in paraffin, 3-μm sections were stained with Meyer's hematoxylin and eosin reagents. For immunohistochemical staining, 4-μm kidney sections on slides were deparaffinized with Histoclear (National Diagnostics, Atlanta, GA) and rehydrated with graded ethanol. After treatment with 0.05% saponin in ddH_2_O, the sections were blocked with 10% normal goat sera for 30 minutes at room temperature. Primary anti-Nek1 antibodies were added at a concentration of 3μg/ml and incubated overnight at 4°C. After extended wash with phosphate-buffered saline (PBS), biotinylated secondary anti-rabbit IgG antibodies and peroxidase-based ABC development kits (Vector Laboratories, Burlingame, CA) were applied. Color was developed and was then counterstained with methyl green to identify nuclei. Our Institutional Review Board specifically approved the use of excess, discarded biopsy or nephrectomy tissues from humans.

### Ionizing and ultraviolet radiation

Cells were γ-irradiated using cesium-40 at the rate of 116 cGy/min. For UV irradiation, cells were first washed with PBS twice and then placed inside a UV cross-linker (Stratagene, La Jolla, CA). The dose of UV irradiation was monitored with a UV meter.

### Chemicals

H_2_O_2_ and the chemotherapeutic drugs (methylmethane sulfonate, etoposide, and 5-FU, cycloheximide) were purchased from Sigma-Aldrich (St. Louis, MO) and reconstituted according to the manufacturer's instructions immediately before addition to cultured cells.

### Adenovirus-Based RNAi Vector Construction

The adenovirus-based RNAi vector was generated by subcloning the transcriptional unit of U6 promoter-Nek1 or -luciferase short hairpin RNA (shRNA) (0.4 kb) into pAdTrack plasmid upstream of the CMV-GFP cassette (1.6 kb). The short inhibitory RNA (shRNA) sequences specific for human Nek1 is 5'-GGAGAGAAGUUGCAGUAUUG-3' and for an irrelevant control gene (firefly Luciferase), it is 5'- AAGAUUCAAAGUGCGCUGCUG –3'. Adenoviruses were generated using an AdEasy Vector System (Stratagene, La Jolla, CA). Briefly, after linearizing with *PmeI*, pShuttle- Nekli CMV-GFP was mixed with pAdEasy-1 and electroporated into competent *E. coli* BJ5183 cells. A recombinant Ad-Nek1i plasmid was obtained, purified, and linearized with *PacI* to transfect into 293 cells. Recombinant Ad-Nek1i adenovirus was then generated, amplified, and titered for further infections. Multiplicities of infection of approximately 30 viral particles per cell were used to obtain efficient gene transduction in all cases using the recombinant adenoviruses, and resulted in >99% of the cells expressing GFP.

### Assays of cell death

Trypan blue exclusion was used to count for viable cell. Staining of nuclei with 4', 6-diamidino-2-phenylindole (DAPI) (1 μg/ml) was also used in individual cells under fluorescence microscopy. Nuclei in dead cells (condensed or fragmented nuclei) could clearly and reproducibly be distinguished from living cells (normal).

### Genotoxic treatment

Cells were treated with MMS at either 0.01% (W/V) or 0.075% (W/V) for one hour. After an hour of treatment, MMS was neutralized by sodium thiosulfate and cells were washed twice with PBS before they were re-fed with fresh media. For gamma irradiation, cells grown in log phase were irradiated with measured doses of γ-rays using cesium-40 at the rate of 116 cGy/min. Medium was replaced for all cells immediately after irradiation. Percentages of cells still surviving 24 hours after different doses of IR were determined by counting the number of cells excluding trypan blue vital dye in triplicates, divided by the total number of cells per plate. For the H_2_O_2_ treatment, H_2_O_2_ was added to the final indicated concentration and cells were cultured for one hour before they were harvested for the analysis. For the 5FU and etoposide treatment, cells were incubated in the indicated concentration of drug for one hour, the drug treatment was then removed and refed with fresh media. 24 hours later, cells were harvested for further analysis.

### Protein stability assay

Cells were treated with cycloheximide (100ug/ml) for the indicated time. At the end of each time point, cells were washed three times with cold 1XPBS. The cell lysate were then prepared, separated by SDS-PAGE and analyzed by for Western Blot for Nek1, CDT1 and tubulin expression.
